# Protective efficacy of multiepitope vaccines constructed from common antigens of *Eimeria* species in chickens

**DOI:** 10.1186/s13567-023-01253-y

**Published:** 2023-12-13

**Authors:** Chen Chen, Junzhi Su, Mingmin Lu, Lixin Xu, Ruofeng Yan, Xiangrui Li, Xiaokai Song

**Affiliations:** MOE Joint International Research Laboratory of Animal Health and Food Safety, College of Veterinary Medicine, Nanjing Agricultural University, Nanjing, 210095 China

**Keywords:** Epitope vaccine, bioinformatics analysis, common antigen, cross-protection, coccidiosis

## Abstract

Clinical avian coccidiosis is typically caused by coinfection with several *Eimeria* species. Recombinant protein and DNA vaccines have shown promise in controlling coccidiosis. On this basis, DNA vaccines that encode multiple epitopes from different *Eimeria* species may provide broad protection against coinfections. In this study, we designed a fusion gene fragment, 14EGT, that contained concentrated T-cell epitopes from four common antigens of *Eimeria* species (14-3-3, elongation factor 2, glyceraldehyde-3-phosphate dehydrogenase, and transhydrogenase). The multiepitope DNA vaccine pVAX1-14EGT and recombinant protein vaccine pET-32a-14EGT (r14EGT) were then created based on the 14EGT fragment. Subsequently, cellular and humoral immune responses were measured in vaccinated chickens. Vaccination-challenge trials were also conducted, where the birds were vaccinated with the 14EGT preparations and later exposed to single or multiple *Eimeria* species to evaluate the protective efficacy of the vaccines. According to the results, vaccination with 14EGT preparations effectively increased the proportions of CD4^+^ and CD8^+^ T cells and the levels of Th1 and Th2 hallmark cytokines. The levels of serum IgG antibodies were also significantly increased. Animal vaccination trials revealed alleviated enteric lesions, weight loss, and oocyst output compared to those of the control groups. The preparations were found to be moderately effective against single *Eimeria* species, with the anticoccidial index (ACI) ranging from 160 to 180. However, after challenge with multiple *Eimeria* species, the protection provided by the 14EGT preparations was not satisfactory, with ACI values of 142.18 and 146.41. Collectively, the results suggest that a multiepitope vaccine that encodes the T-cell epitopes of common antigens derived from *Eimeria* parasites could be a potential and effective strategy to control avian coccidiosis.

**Supplementary Information:**

The online version contains supplementary material available at 10.1186/s13567-023-01253-y.

## Introduction

Avian coccidiosis, an important disease, poses obstacles to the development of the poultry industry. Clinical coccidiosis typically causes malabsorption, impaired feed conversion, and various forms of enteritis. In severe cases, this condition can even lead to death. The global economic impact of avian coccidiosis infections in 2016 was substantial, with an estimated loss of £10.4 billion [1]. Losses due to prophylactic control, therapeutic treatment, morbidity, and mortality constitute the primary components of the overall economic impact. For effective management of coccidiosis, conventional approaches involve employing anticoccidial drugs and implementing vaccination methods. Nevertheless, the usage of coccidiostats is often linked with the development of drug resistance. Increasing consumer awareness of food safety has also prompted many government agencies worldwide to prohibit farmers from administering anticoccidial drugs [2]. Vaccination is considered a more desirable approach for widespread clinical use than coccidiostats. However, traditional live vaccines are becoming increasingly challenging to implement due to their inherent drawbacks when compared to novel anticoccidial vaccines [3]. Recombinant protein and DNA vaccines have been shown to have important roles in controlling chicken coccidiosis. For example, CoxAbic^®^ (Netanya, Israel), the only commercial subunit vaccine against coccidiosis, can protect broiler offspring from coccidiosis through maternal antibodies produced by vaccinated laying chickens [4]. DNA vaccines incorporating genes from *Eimeria* pathogens have also demonstrated excellent protective efficacy against infection by these species [5–7]. Recently, multiepitope DNA vaccines have emerged as a promising strategy for combating parasitic infections and have garnered widespread recognition for their efficacy. For instance, in a study involving BALB/c mice with toxoplasmosis, major improvements in parasite burden and survival time were observed following subcutaneous vaccination with a multiepitope DNA vaccine encoding SAG1, SAG3, and SAG5 proteins from *Toxoplasma gondii* [8]. Likewise, a multiepitope DNA vaccine composed of T- and B-cell epitopes of rhoptry protein 8 (ROP8) from *T. gondii* has been shown to increase the survival time of infected BALB/c mice and stimulate robust T and B-cell responses [9]. Regarding the control of coccidiosis, Song et al. identified concentrated T-cell epitope fragments from merozoite antigens MZ5-7 and sporozoite antigen SO7 of *Eimeria tenella* [10]. Subsequent challenge trials demonstrated that the vaccine constructed using these epitope fragments had excellent protective efficacy. Therefore, developing multiepitope vaccines containing T- or B-cell epitopes from parasitic antigens may be an effective strategy for addressing the growing threat of coccidiosis.

Numerous studies examining the prevalence of *Eimeria* species across various regions have consistently found that clinical avian coccidiosis is typically caused by multiple *Eimeria* species [11–13]. Host immunity against *Eimeria* is known to be species-specific [14]. As a result, vaccines designed to target parasitic antigens from only a single *Eimeria* species may not provide chickens with sufficient protection in real-world settings. For an anticoccidial vaccine to be truly effective and successful, it should include antigens that are capable of inducing host immune responses against different *Eimeria* species. In other words, the vaccine should encompass the antigens shared by multiple *Eimeria* species [15], and such antigens have been characterized in several studies. Talebi, for example, identified a conserved protein band (45 kDa) by comparing the expressed protein of sporulated oocysts across five major *Eimeria* species [16]. Additionally, Sasai et al. and Constantinoiu et al. each identified highly conserved antigens located on the apical complex of *Eimeria* sporozoites [17, 18]. However, it should be noted that these antigens mentioned above are not specific to any particular *Eimeria* species, and their efficacy has not been confirmed by animal protection experiments. Recently, our laboratory conducted an immunoproteomic analysis to screen five common antigens that exhibited high similarity (more than 90%) across *Eimeria tenella*, *E. maxima*, and *E. acervulina* [19]. Two of them have been confirmed to provide adequate protection against various *Eimeria* species [20, 21]. In this study, we selected four common antigens, namely, 14-3-3, glyceraldehyde-3-phosphate dehydrogenase (GAPDH), transhydrogenase, and elongation factor 2 (EF-2), to develop a multiepitope DNA vaccine. The ultimate goal herein is to discover a robust strategy for controlling the transmission of coccidiosis.

Cell-mediated immune responses, specifically T-cell-mediated immune responses, are recognized as pivotal in combating infections caused by *Eimeria* species [22, 23]. Upon activation by invading pathogens, CD4^+^ T cells can differentiate into various types of helper T cells that are responsible for regulating host immune responses by secreting multiple cytokines. For instance, Lillehoj et al. discovered the inhibitory effect of recombinant IFN-γ on the in vitro development of *Eimeria* species and its potential role in reducing oocyst output [24]. Cytotoxic T cells, which are derived from CD8^+^ T cells, are capable of specifically targeting and killing enterocytes that have been invaded by *Eimeria* species [25]. In addition, coccidia can employ immune evasion strategies by stimulating the production of regulatory T cells (Tregs) [26]. The critical roles played by T cells during coccidiosis highlight the strong potential application of T-cell epitopes derived from parasitic antigens in the development of multiepitope vaccines against coccidiosis. To this end, we screened gene fragments containing concentrated T-cell epitopes from the four common antigens of *Eimeria* parasites and generated a combined gene fragment, 14EGT. The multiepitope DNA vaccine and recombinant proteins were produced based on the 14EGT fragment. As such, we herein aimed to establish a new control strategy for avian coccidiosis while also serving as a source of inspiration for managing coinfection with other pathogens.

## Materials and methods

### Animals, parasites, and reagents

Newborn Hy-Line white chickens, aged 1 day old, were raised in sanitary rooms without coccidia contamination throughout the experiment. All flocks were provided ad libitum access to food and water without any coccidiostats. The eukaryotic expression vector pVAX1 and the prokaryotic expression vector pET-32a were purchased from Novagen (Darmstadt, Germany). HEK 293 T cells and chicken serum against *E. tenella*, *E. maxima*, and *E. acervulina* were provided by our laboratory. Mouse anti-chicken CD3-FITC, mouse anti-chicken CD4-PE, and mouse anti-chicken CD8-PE fluorescent antibodies were purchased from Southern Biotechnology Associates (Birmingham, AL, USA). Sporulated oocysts of *E. tenella*, *E. maxima,* and *E. acervulina* were stored in 2.5% potassium dichromate at 4 ℃. Oocyst rejuvenation was conducted using healthy chickens every three months. Female SD rats were purchased from the Qinglongshan animal breeding farm in Nanjing, China. The ethical and welfare aspects of all animal studies and experimental protocols conducted during the research were duly approved by the Committee on Experimental Animal Welfare and Ethics at Nanjing Agricultural University.

### Analysis and screening of T-cell epitopes from *Eimeria* common antigens

According to the accession numbers of these common antigens, the amino acid sequences of 14-3-3 (XP_013250285.1), elongation factor 2 (XP_013332773.1), GAPDH (XP_013251305.1) and transhydrogenase (XP_013252758.1) were retrieved from NCBI. The analysis of the T-cell epitope was conducted using DNAStar Protean (version 7.1.0, DNASTAR, Inc., Madison, WI, USA), following the approach described by Berzofsky et al., Rothbard and Taylor, and Jameson and Wolf [27–29]. Hopp-Woods’s method was used to identify the hydrophilic regions of four common antigens [30]. The potential T-cell epitope amino acid sequences were selected by analysing high antigenic index along with hydrophilicity. The selected sequences were analysed for their binding sites to MHC I and MHC II molecules using NetMHC, which includes NetMHCcons 1.1 Server and NetMHCIIpan 4.0 Server. Notably, the prediction of avian antigen epitope binding to MHC molecules must be performed using an indirect method. For example, for prediction of the binding of the same 9 amino acid fragment that coexists in chicken MHC I and alleles of human MHC I molecules, the chicken MHC I molecule was replaced with three alleles of the human MHC I molecule (HLA-B 40:06, HLA-B 41:04, and HLA-B 41:03) [31]. Analysis of binding sites between common antigens and chicken MHC I using NetMHCcons 1.1 Server was carried out based on the threshold IC50 value for binding affinity. Similarly, the prediction of binding sites between the targeted antigens and chicken MHC II was initially performed by screening the same 15-amino acid fragment present in four alleles of human MHC II molecules (DRB1:1482, DRB1:1366, DRB1:1310, and DRB1:1445). Then, the screening of binding amino acid sequences was conducted using NetMHCIIpan 4.0 Server [32]. Table 1 provides a comprehensive list of relevant indices and a detailed description of the screening process.

**Table 1:** **Bioinformatics analysis of four common antigens**.

Gene	Fragment	Size (aa)	Prediction of the binding site to MHC I							Prediction of the binding site to MHC II							
			Allele	Position	Epitope	Score (aff)	IC50 (nm)	%Rank^a^	Binding Level^b^	Allele	Position	Epitope	Core	Score-EL^c^	%Rank-EL^d^	Score-BA^e^	%Rank-BA^f^
14-3-3	14	149 (95–243)	HLA-B40:06	178	AELPSTHPI	0.511	198.69	0.15	≤ SB	DRB1-1310	145	YYRYISEFSNEEGKK	YISEFSNEE	0.659719	0.87 (SB)	0.368476	2.88
											144	DYYRYISEFSNEEGK	YISEFSNEE	0.575921	1.50 (SB)	0.351085	4.26
			HLA-B41:03	178	AELPSTHPI	0.668	36.31	0.12	≤ SB		143	GDYYRYISEFSNEEG	YRYISEFSN	0.555038	1.72 (SB)	0.336931	5.73
										DRB1-1366	145	YYRYISEFSNEEGKK	YISEFSNEE	0.518796	1.27 (SB)	0.388953	11.89
			HLA-B41:04	178	AELPSTHPI	0.678	32.48	0.05	≤ SB	DRB1-1445	95	NKALAASYRQKVENE	LAASYRQKV	0.663603	0.22 (SB)	0.346217	4.48
										DRB1-1482	95	NKALAASYRQKVENE	LAASYRQKV	0.792532	0.13 (SB)	0.511766	2.99
EF2	E	177 (387–563)	HLA-B40:06	524	GELHVEICL	0.372	888.54	0.80	≤ WB	DRB1-1310	476	SPVVRVAVKPKDMKE	VRVAVKPKD	0.895005	0.05 (SB)	0.368398	2.88
											541	QIDIIVSDPVVSYRE	IVSDPVVSY	0.577648	1.48 (SB)	0.399450	1.37
										DRB1-1366	476	SPVVRVAVKPKDMKE	VRVAVKPKD	0.855490	0.06 (SB)	0.419370	7.71
			HLA-B41:03	524	GELHVEICL	0.591	83.09	0.50	≤ SB		541	QIDIIVSDPVVSYRE	IVSDPVVSY	0.527570	1.21 (SB)	0.523473	1.06
										DRB1-1445	476	SPVVRVAVKPKDMKE	VRVAVKPKD	0.756613	0.04 (SB)	0.312353	8.66
			HLA-B41:04	524	GELHVEICL	0.457	356.37	0.80	≤ WB		541	QIDIIVSDPVVSYRE	IVSDPVVSY	0.704201	0.10 (SB) 0.453546 0.29	0.453546	0.29
										DRB1-1482	476	SPVVRVAVKPKDMKE	VRVAVKPKD	0.763648 0.785701	0.19 (SB) 0.14(SB)	0.432076	12.81
											541	QIDIIVSDPVVSYRE	IVSDPVVSY	0.785701	0.14 (SB)	0.600302	0.23
GAPDH	G	176 (52–227)	HLA-B40:06	138	EEYQPTLQV	0.371	902.73	0.80	≤ WB	DRB1-1310	115	AKKVIISAPPKDDTP	VIISAPPKD	0.703120	0.62 (SB)	0.318953	8.03
											128	TPMFVMGVNHEEYQP	FVMGVNHEE	0.597728	1.31 (SB)	0.361971	3.35
			HLA-B41:03	138	EEYQPTLQV	0.491	247.81	1.50	≤ WB		63	DGNLVVEGKTIQVFA	LVVEGKTIQ	0.487992	2.60 (WB)	0.283325	15.25
										DRB1-1366	115	AKKVIISAPPKDDTP	VIISAPPKD	0.576140	0.89 (SB)	0.365268	16.19
				68	VEGKTIQVF	0.548	133.03	1.00	≤ WB		63	DGNLVVEGKTIQVFA	LVVEGKTIQ	0.475566	1.62 (SB)	0.389848	11.76
										DRB1-1445	63	DGNLVVEGKTIQVFA	LVVEGKTIQ	0.534977	0.87 (SB)	0.330942	6.06
			HLA-B41:04	104	KEKAGLHIS	0.355	1069.33	2.00	≤ WB		115	AKKVIISAPPKDDTP	VIISAPPKD	0.485379	1.33 (SB)	0.267099	18.43
											205	GSNIIPASTGAAKAV	IIPASTGAA	0.436942	1.95 (SB)	0.320841	7.38
				68	VEGKTIQVF	0.346	1182.43	2.00	≤ WB	DRB1-1482	115	AKKVIISAPPKDDTP	VIISAPPKD	0.579515	1.13 (SB)	0.406430	18.20
											63	DGNLVVEGKTIQVFA	LVVEGKTIQ	0.501950	1.91 (SB)	0.457851	8.44
Transhydrogenase	T	326 (579–904)	HLA-B40:06	646	AEVLLRVS	0.527	166.85	0.12	≤ SB	DRB1_1310	811	GDAYQRAQRELIANT	YQRAQRELI	0.701329	0.63 (SB)	0.390542	1.71
											890	VDGITVIGRKRIETR	ITVIGRKRI	0.626872	1.10 (SB)	0.355965	3.84
				743	VEAAKVFVI	0.485	264.37	0.20	≤ SB	DRB1_1366	811	GDAYQRAQRELIANT	YQRAQRELI	0.532553	1.18 (SB)	0.444541	5.14
											890	VDGITVIGRKRIETR	ITVIGRKRI	0.513415	1.30 (SB)	0.462875	3.71
			HLA-B41:03	743	VEAAKVFVI	0.659	40.24	0.15	≤ SB	DRB1_1445	890	VDGITVIGRKRIETR	ITVIGRKRI	0.821916	0.00 (SB)	0.413373	0.87
											772	FGHDVRSATREEVES	VRSATREEV	0.699963	0.11 (SB)	0.275680	16.11
											811	GDAYQRAQRELIANT	YQRAQRELI	0.419602	2.23 (WB)	0.275648	16.12
			HLA-B41:04	646	AEVLLRVSA	0.602	74.56	0.15	≤ SB	DRB1_1482	890	VDGITVIGRKRIETR	ITVIGRKRI	0.872567	0.02 (SB)	0.544946	1.38
											772	FGHDVRSATREEVES	VRSATREEV	0.822842	0.08 (SB)	0.406524	18.18
				743	VEAAKVFVI	0.589	85.54	0.17	≤ SB		698	VPRVTRAQKLDVKSA	VTRAQKLDV	0.737749	0.27 (SB)	0.544297	1.40
											811	GDAYQRAQRELIANT	YQRAQRELI	0.551109	1.37 (SB)	0.446054	10.27

**a**, Ranking of predicted scores of selected epitopes among a group of 200 000 random natural 9 aa short peptides. b, Determination of the binding strength between selected epitopes and MHC molecules. The selected epitopes will be identified as strong binders (SBs) if %Rank < 0.5 or IC50 < 50. The selected epitopes will be identified as weak binders (WBs) if 0.5 < %Rank < 2 or 50 < IC50 < 500. c, Predictiveness scores of the screened epitopes. d, In a group of random natural peptides, the percentile rank of the predictiveness scores of the screened epitopes. The selected epitopes will be identified as strong binders (SBs) if %Rank-EL < 2. The selected epitopes will be identified as strong binders (SBs) if 2 < %Rank-EL < 5. e, The predicted binding affinity in log-scale (printed only if binding affinity predictions were selected). f, percentile rank of predicted affinity compared to a group of 100 000 random natural peptides. This measure is not affected by inherent bias of certain molecules towards higher or lower mean predicted affinities (printed only if binding affinity predictions were selected).

### Amplification of the selected T-cell epitope fragments

The total RNA of sporulated oocysts from *E. maxima* and *E. acervulina* was extracted using the Total RNA Kit I (OMEGA, Norcross, Georgia) following the manufacturer’s instructions. Complementary DNA (cDNA) was synthesized from the extracted total RNA using reverse transcription polymerase chain reaction (RT‒PCR). The primers used to amplify the selected gene fragments were designed using Premier Primer 5 software (Premier, Canada), as shown in Table 2. The PCR amplification procedure was performed as follows: a predenaturation step at 95 ℃ for 3 min, followed by 33 cycles of denaturation at 95 ℃ for 15 s, annealing at 58 ℃ for 15 s, and extension at 72 ℃ for 1 min, followed by a final extension step at 72 ℃ for 5 min. The identified target genes were purified and recycled using agarose gel electrophoresis and the Gel Extraction Kit D2500 (OMEGA, Norcross, Georgia), respectively.

**Table 2:** **Primers for gene amplification of four common antigens**.

Source	Gene	Fragment	Primer	Restriction enzyme	Sequence (5ʹ to 3ʹ)	Accession No
*E. acervulina*	14–3-3	14	Forward (no ATG)	*Kpn*I	CGGGGTACCAACAAAGCACTTGCAGCTAGCT	XM_013394831.1
			Forward (with ATG)	*Kpn*I	CGGGGTACCATGAACAAAGCACTTGCAGCTAGCT	
			Reverse	*Hin*dIII	CCCAAGCTTGTCGCGCAACAGCTGCA	
	GAPDH	G	Forward	*Not*I	ATTTGCGGCCGCTCACGGCAAATTCCCTGG	XM_013395851.1
			Reverse	*Bam*HI	CGCGGATCCGTTAAGAGAAGGAATAACTTTCCCT	
	Transhydrogenase	T	Forward	*Bam*HI	CGCGGATCCAGATTGGCTGTTGGTGTTCT	XM_013397304.1
			Reverse (no TAA)	*Xho*I	CCGCTCGAGGCGGGTTTCAATGCGT	
			Reverse (with TAA)	*Xho*I	CCGCTCGAGTAAGCGGGTTTCAATGCGT	
*E. maxima*	EF-2	E	Forward	*Hin*dIII	CCCAAGCTTTTCGGTCGTGTGTTCTCTG	XM_013477319.1
			Reverse	*Not*I	ATTTGCGGCCGCCATGGACGAGGGAGCG	

The restriction enzyme sites are underlined

These primers have been confirmed to serve as cDNA primers for their corresponding genes. Primers with the initiator codon ATG and stop codon TAA were used in the construction of the eukaryotic expression plasmid pVAX1-14EGT, while primers without the initiator codon ATG and stop codon TAA were used in the construction of the prokaryotic expression plasmid pET-32a-14EGT.

### Construction of the multiepitope DNA vaccine pVAX1-14EGT

The recycled gene fragments Ea14-3-3 (14) and EmEF2 (E) were digested with the corresponding restriction enzyme and ligated into the pVAX1 expression vector to produce the recombinant plasmid pVAX1-14E. Endonuclease digestion and sequencing analysis were conducted to identify the constructed recombinant plasmid. Likewise, the T-cell epitope fragments of EaGAPDH (G) and EaTranshydrogenase (T) were ligated into the recombinant plasmid pVAX1-14E using the same procedure to construct the multiepitope DNA vaccine pVAX1-14EGT. Sequence analysis was conducted using a basic alignment search tool on NCBI (BLAST). The prokaryotic expression plasmid pET-32a-14EGT was generated following the methods described above. Notably, the primers used in this study did not contain the initiation codon ATG or the stop codon TAA.

### Generation of the purified 14EGT recombinant protein and its antiserum

For production of r14EGT, the pET-32a-14EGT plasmid was first transformed into *E. coli* BL21. The expression of r14EGT was then induced by adding isopropyl-beta-D-thiogalactopyranoside (IPTG), resulting in a high level of protein expression. The expressed recombinant protein was confirmed using SDS‒PAGE analysis. For purification of r14EGT, a Ni–NTA column (GE Healthcare, USA) was utilized, followed by endotoxin removal using the ToxinEraserTM Endotoxin Removal Kit (GenScript, China).

Healthy and disease-free female SD rats were used to prepare anti-r14EGT rat serum. For the primary immunization, the rats were subcutaneously injected in the back with 200 μg of r14EGT mixed with an equal volume of Freund’s complete adjuvant (Sigma‒Aldrich, Darmstadt, Germany). After 14 days, the same immunization procedure as the primary immunization was performed for the booster immunization. However, Freund’s complete adjuvant was replaced with Freund’s incomplete adjuvant (Sigma‒Aldrich, Darmstadt, Germany) for booster immunization. Following the booster immunization, the 3rd and 4th immunizations were conducted using the same method. 10 days after the rats received the fourth booster immunization, blood was collected from the abdominal aorta to prepare antiserum. The antiserum that had been prepared was stored at −80 ℃ for future use. Moreover, serum was collected from rats that had not been exposed to the antigen as a negative control.

### Recognition of recombinant protein 14EGT by immunoblot assays

Western blot assays were performed to determine the expression of r14EGT. In brief, purified r14EGT was separated using SDS‒PAGE and then transferred onto polyvinylidene difluoride (PVDF) membranes. The membranes were then blocked with 5% skim milk powder diluted in Tris-buffered saline containing 0.5% Tween-20 (TBST) at 37 ℃ for 2 h, after which the membranes were washed with TBST. The membranes were incubated with primary antibodies diluted in TBST (1:100 for chicken sera anti-*E. tenella*, *E. maxima*, or *E. acervulina*, and 1:10 000 for His-tag mouse monoclonal antibody) at 4 ℃ overnight. After five washes with TBST, the membranes were incubated with HRP-labelled secondary antibodies diluted in TBST (1:20 000 for goat anti-chicken IgG antibody or 1:10 000 for goat anti-mouse IgG antibody) for 1 h at 37 ℃. For analysis of the reaction, the membranes were subjected to colour development using ECL chemiluminescence detection from Tiangen Biotech (Beijing, China). A negative control was employed using healthy chicken serum as the primary antibody.

### Detection of pVAX1-14EGT expression in eukaryotic cells

Prior to administration of the multiepitope DNA vaccine pVAX1-14EGT to chickens, its eukaryotic expression in HEK 293 T cells was evaluated. The eukaryotic expression plasmids pVAX1-14EGT and pVAX1 empty plasmid were transfected separately into HEK 293 T cells using the LipofectamineTM 3000 kit (Thermo Fisher Scientific, Waltham, MA, USA) according to the manufacturer’s instructions. The transfected cells were washed with sterile phosphate-buffered saline (PBS) and fixed with 4% paraformaldehyde at 4 ℃ overnight. After three washes with TBST, the fixed cells were blocked with 5% bovine serum albumin (BSA) diluted in TBST at 37 ℃ for 1 h. The cells were subjected to an antigen–antibody reaction with rat serum against r14EGT diluted in 5% BSA at a 1:100 dilution at 4 ℃ overnight. Normal rat serum was used as the primary antibody for the control group. The cells transfected with pVAX1-14EGT and incubated with anti-r14EGT rat serum were designated the pVAX1/14EGT + group. The cells transfected with the pVAX1 plasmid and incubated with anti-r14EGT rat serum were designated the pVAX1 + group. The cells transfected with pVAX1-14EGT and incubated with negative rat serum were designated the pVAX1/14EGT group. Finally, the cells transfected with the pVAX1 plasmid and incubated with negative rat serum were designated the pVAX1 group. After three washes with TBST, the slides were incubated with Cy3-conjugated goat anti-rat IgG antibody (diluted 1:1000) in the dark at 37 °C for 1 h. After the secondary antibody was washed away with TBST, the slides were stained with 4ʹ,6-diamidino-2-phenylindole (DAPI, Beyotime, Shanghai, China) at a 10 μg/mL concentration in PBS. The slides were examined using a fluorescence microscope (Carl Zeiss, Jena, Germany).

### Animal immunization and splenocyte isolation

At 14 days of age, a group of healthy chickens was randomly divided into five groups, with each group consisting of six chickens. The chickens were then immunized intramuscularly with their appropriate preparations. Specifically, the experimental groups comprised chickens immunized with pVAX1-14EGT (100 μg/chicken) and r14EGT (200 μg/chicken). The chickens in the control groups were treated with pVAX1 empty vector (100 μg/chicken), pET-32a tag protein (200 μg/chicken), or PBS. After seven days, a booster immunization was carried out using the same procedure as the primary immunization.

Three chickens were randomly selected from each group and humanely sacrificed seven days after the primary and booster immunizations to prepare splenocytes. In brief, the spleens were removed and completely ground using the handle of a 20 mL syringe. The ground spleens were then mixed with 5 mL of PBS and filtered through 200 mesh cell strainers. The filtrate was carefully added to 15 mL centrifuge tubes containing 5 mL of lymphocyte separation solution (TBD, Tianjin, China). After centrifugation at 2000 rpm for 10 min, the white annular splenocytes were transferred to a new 15 mL centrifuge tube. The splenocytes were then subjected to two rounds of centrifugation and washed twice with PBS. The concentration of the splenocytes was adjusted to 1 × 10^7^ cells/mL using PBS.

### Detection of cellular immune responses in chickens induced by vaccination

Ratios of CD4^+^ and CD8^+^ T cells from splenocytes were measured using flow cytometry. Briefly, 1 × 10^6^ splenocytes (100 μL) from each chicken in the experimental groups were added to 2 mL centrifuge tubes. Dual staining was performed on the splenocytes using 2 μL of mouse anti-chicken CD3 antibody mixed with an equal volume of mouse anti-chicken CD4 antibody or with 2 μL of mouse anti-chicken CD3 antibody mixed with an equal volume of mouse anti-chicken CD8 antibody. Splenocytes prepared from the PBS control group were stained as follows: three samples were stained with mouse anti-chicken CD3, CD4, and CD8 antibodies, and one sample was left unstained as a negative control. All samples were incubated with the corresponding antibody in darkness for 30 min. The stained splenocytes were then washed with PBS before being analysed using a FACSCalibur flow cytometer (BD Biosciences, Franklin Lakes, NJ, USA).

The expression levels of Th1 and Th2 cytokines (IL-2, IL-4, and IFN-γ) were quantified in splenocytes collected from immunized chickens using qRT‒PCR. The extraction of total RNA from prepared splenocytes was carried out according to the manufacturer’s instructions. The cytokine amplification primers can be found in Table 3. In this trial, the *GAPDH* gene served as an internal control, as previously described [33]. The 2^−∆∆CT^ method was utilized to measure the relative expression levels of cytokines in the treated groups compared to the internal control (represented as n-fold changes relative to the water control group) [34].

**Table 3:** **Primers used in qPCR**.

RNA target	Primer sequence	Accession No.
GAPDH	GGTGGTGCTAAGCGTGTTAT ACCTCTGTCATCTCTCCACA	K01458
IL-2	TAACTGGGACACTGCCATGA GATGATAGAGATGCTCCATAAGCTG	AF000631
IL-4	ACCCAGGGCATCCAGAAG CAGTGCCGGCAAGAAGTT	AJ621735
IFN-γ	AGCTGACGGTGGACCTATTATT GGCTTTGCGCTGGATTC	Y07922

### Detection of specific antibody IgG in serum from vaccinated chickens

On the seventh day of primary and booster immunization, serum was collected randomly from three chickens in each group. An indirect enzyme-linked immunosorbent assay (ELISA) was then performed to measure the levels of specific IgG antibodies in the serum of immunized chickens. Briefly, the concentration of purified r14EGT was adjusted to 10 μg/mL using 0.05 M carbonate buffer. The diluted recombinant protein (200 μL) was coated onto flat-bottomed 96-well plates and incubated at 4 ℃ for 16 h. After being washed with PBST, the plates were blocked with 5% BSA at 37 ℃ for 2 h. After blocking, the plates were washed with PBST and then incubated with prepared chicken serum as the primary antibody (diluted with 5% BSA, 1:50) at 37 ℃ for 1 h. The primary antibody of the negative control and blank control was replaced by an equal volume of naïve chicken serum and PBS, respectively. After the incubation was complete, the plates were washed thoroughly, and HRP-conjugated goat anti-chicken IgG antibody was added to each well as the secondary antibody. The antibody was diluted with 5% BSA at a ratio of 1:40 000. After a 45 min incubation with primary and secondary antibodies at 37 ℃, any unbound antibodies were removed by washing with PBST. The colour development was performed by adding 100 μL of 3,3ʹ,5,5ʹ-tetramethylbenzidine (TMB) to the samples and incubating them in the dark at room temperature for 8 min. The reactions were then determined using a spectrophotometric method at a wavelength of 450 nm (OD450).

### Protective efficacy evaluation of 14EGT vaccines against challenge infection by *Eimeria* species

The protective efficacy of 14EGT vaccines against *E. tenella*, *E. maxima*, and *E. acervulina*, as well as mixed infections of these three *Eimeria* species, was evaluated in four vaccination-challenge trials (Table 4). Trials 1–4 were designed to evaluate the protective efficacies of the vaccines against challenges posed by *E. maxima* (Trial 1), *E. acervulina* (Trial 2), *E. tenella* (Trial 3), and mixed infections by the aforementioned three *Eimeria* species (Trial 4). Fourteen-day-old chicks with similar body weights were used in the trials. Each trial consisted of six groups, with each group comprising 15 chicks that were 14 days old. Trials 1 and 2 used the data from the same unchallenged control, and likewise for Trials 3 and 4. Experimental groups were intramuscularly immunized in the legs with pVAX1-14EGT (100 μg/chicken) or r14EGT (200 μg/chicken). The control groups received different injections: 100 μg of pVAX1 empty plasmid per chicken for the pVAX1 controls, 200 μg of pET-32a tag protein per chicken for the pET-32a tag protein controls, and PBS for both the unchallenged controls and challenged controls. All injections were administered into the leg muscles. On the seventh day after the primary immunizations, booster immunizations were administered using the same procedure as the primary immunizations. At 28 days old, each chicken (all groups) was weighed and orally challenged (except for the unchallenged group) with either 1 × 10^5^ *E. maxima* in trial 1, 1 × 10^5^ *E. acervulina* in trial 2, 5 × 10^4^ *E. tenella* in trial 3, or a mixture of oocysts consisting of 1 × 10^5^ *E. maxima*, 1 × 10^5^ *E. acervulina*, and 5 × 10^4^ *E. tenella* in trial 4. Seven days after the challenge, all chickens were weighed and then slaughtered to obtain raw data on body weight gain, oocyst shedding, enteric lesion score, and anticoccidial index (ACI). The rate of weight gain was calculated using the following formula: rate of weight gain = (mean weight before slaughter—mean weight before challenge)/(mean weight before challenge) × 100%. The relative rate of weight gain was calculated using the following formula: (relative rate of weight gain) = (rate of weight gain of experimental groups)/(mean weight on the day of slaughter of the unchallenged control) × 100%. Oocyst counting was performed using a McMaster chamber following the method described by Hodgson in 1970 [35]. A numerical scale ranging from 0 to 4 was used to score the severity of enteric lesions, following an established method [36]. The oocyst shedding decrease ratio was calculated using a previously reported formula [37]. The ACI was calculated according to the following formula: (relative rate of weight gain + survival rate) − (lesion index + oocyst index) [38, 39]. The protective efficacy of 14EGT vaccines against coccidiosis was evaluated based on the values of an anticoccidial index (ACI). An ACI value greater than 180 indicates that the vaccine has excellent protective efficacy; an ACI value between 160 and 180 indicates that the vaccine has moderate protective efficacy; an ACI value between 120 and 160 indicates that the vaccine has unsatisfactory protective efficacy; and an ACI value less than 120 indicates that the vaccine has no protective efficacy.

**Table 4:** **Protective efficacies of multiepitope vaccines constructed from common antigens of *Eimeria* species in chickens**.

Trials	Challenged *Eimeria* species	Groups	Weight gain (g)	Mean lesion score	Mean OPG × 10^5^	Oocyst decrease rate %	ACI
Trial 1	*E. maxima*	Unchallenged control*	44.14 ± 8.04^a^	0 ± 0^a^	0 ± 0^a^	100	200
		Challenged control	19.97 ± 5.70^b^	2.27 ± 0.68^bc^	1.49 ± 0.044^b^	0	83.02
		pVAX1 control	23.15 ± 8.35^b^	2.33 ± 0.60^bc^	1.40 ± 0.26^b^	6.26	88.63
		pET-32a tag protein control	23.69 ± 7.61^b^	2.53 ± 0.50^b^	1.10 ± 0.49^c^	26.49	102.4
		pVAX1-14EGT	40.99 ± 11.54^a^	1.93 ± 0.68^ cd^	0.32 ± 0.11^d^	78.83	173.17
		r14EGT	39.78 ± 8.20^a^	1.73 ± 0.57^d^	0.37 ± 0.08^d^	74.76	162.4
Trial 2	*E. acervulina*	Unchallenged control*	44.14 ± 8.04^a^	0 ± 0^a^	0 ± 0^a^	100	200
		Challenged control	27.26 ± 9.02^b^	2 ± 0.73^b^	0.28 ± 0.07^b^	0	102.69
		pVAX1 control	26.19 ± 7.33^b^	1.6 ± 0.49^c^	0.21 ± 0.05^c^	25.24	124.35
		pET-32a tag protein control	26.87 ± 9.34^b^	1.6 ± 0.49^c^	0.19 ± 0.04^c^	34.21	124.96
		pVAX1-14EGT	40.73 ± 7.70^a^	1.4 ± 0.49^c^	0.08 ± 0.01^d^	62.69	169.87
		r14EGT	40.20 ± 11.08^a^	1.27 ± 0.44^c^	0.11 ± 0.02^e^	62.17	168.11
Trial 3	*E. tenella*	Unchallenged control^#^	41.05 ± 16.08^a^	0 ± 0^a^	0 ± 0^a^	100	200
		Challenged control	8.59 ± 6.37^b^	2.53 ± 0.50^b^	6.38 ± 1.03^b^	0	76
		pVAX1 control	14.3 ± 5.81^b^	2.87 ± 0.50^b^	5.91 ± 0.44^bc^	7.4	91.41
		pET-32a tag protein control	11.15 ± 8.31^b^	2.8 ± 0.75^b^	5.41 ± 1.89^bc^	15.28	84.36
		pVAX1-14EGT	37.11 ± 12.18^a^	1.33 ± 0.70^c^	1.42 ± 0.16^d^	77.82	172.79
		r14EGT	35.39 ± 8.23^a^	1.93 ± 0.77^d^	1.15 ± 0.36^d^	82.04	163.8
Trial 4	Mixed infection	Unchallenged control^#^	41.05 ± 16.08^a^	0 ± 0^a^	0 ± 0^a^	100	200
		Challenged control	12.00 ± 9.50^b^	3.33 ± 0.47^b^	14.60 ± 1.96^b^	0	56.14
		pVAX1 control	15.21 ± 8.64^b^	3.67 ± 0.47^b^	13.96 ± 3.02^b^	4.36	46.95
		pET-32a tag protein control	15.49 ± 10.47^b^	3.6 ± 0.49^b^	13.44 ± 5.19^b^	7.89	48.06
		pVAX1-14EGT	32.83 ± 8.52^a^	2.73 ± 0.44^c^	7.00 ± 0.90^c^	52.06	142.18
		r14EGT	33.05 ± 7.88^a^	2.53 ± 0.50^c^	6.51 ± 0.86^c^	55.4	146.41

1. Value = mean ± S.D., *n* = 15. 2. Data comparisons were limited to within each individual trial and were not conducted across the four trials. 3. In the same column within a trial, data superscripts sharing the same letter (a–d) denote a nonsignificant difference (*p* > 0.05), while different letters a–d indicate a significant difference (*p* < 0.05). 4.

*Trial 1 and trial 2 shared the data from the same unchallenged control.

^#^Trial 3 and trial 4 shared the data from the same unchallenged control. 5. Mixed infection: challenge with the mixed oocyst of the above 3 *Eimeria* species (1 × 10^5^ *E. maxima*, 1 × 10^5^ *E. acervulina* and 5 × 10^4^ *E. tenella*).

### Statistical analysis

Statistical analysis was conducted using the SPSS statistical package (SPSS 20, SPSS, Inc., Chicago, IL, USA). The data on the cellular immune response and serum-specific antibodies induced by 14EGT vaccines were analysed by the Kruskal‒Wallis H test with statistical significance set at *p* < 0.05. The data on vaccination-challenge trials were analysed by one-way ANOVA with Duncan’s test with statistical significance set at *p* < 0.05.

## Results

### Selection of T-cell epitope fragments from the genes of *Eimeria* common antigens

The amino acid sequences used for constructing the multiepitope vaccine were analysed using DNAStar Protean software. After taking the density of T-cell epitopes, antigenic index scores, and the hydrophilicity of amino acid sequences into account, we selected the following T-cell epitope fragments: 149 amino acids from *E. acervulina* 14-3-3 (95–243), 177 amino acids from *E. maxima* EF2 (387–563), 176 amino acids from *E. acervulina* GAPDH (52227) and 326 amino acids from *E. acervulina* transhydrogenase (579–904). Additional file 1 presents the relevant prediction results obtained using DNAStar Protean software. The selected amino acid sequences were analysed for their binding sites to MHC I and MHC II molecules using NetMHCcons 1.1 Server and NetMHCIIpan 4.0 Server. Table 1 shows that the selected fragments have binding sites to both MHC I and MHC II molecules, indicating their potential to be used in the construction of multiepitope vaccines.

### Construction of expression plasmids encoding the 14EGT chimeric gene

**Figure 1:**
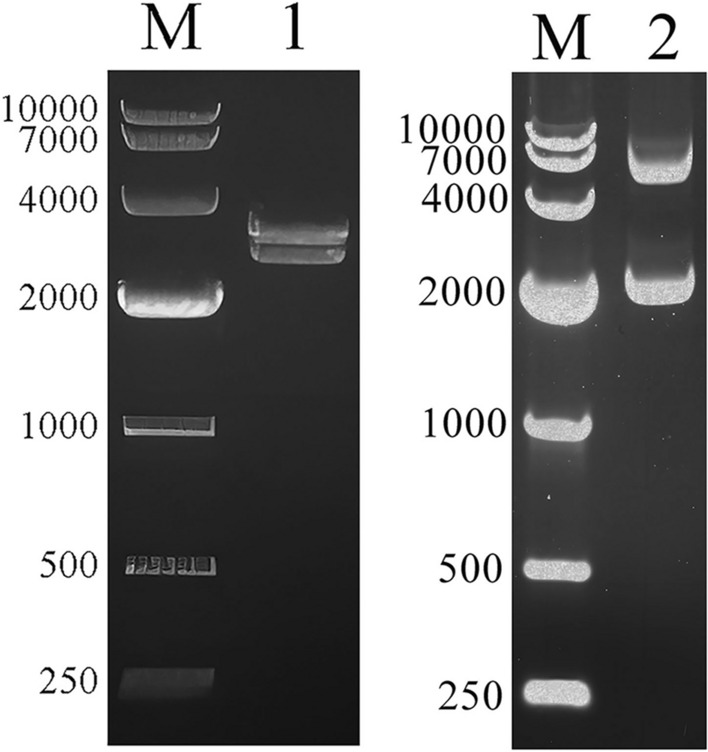
**Endonuclease digestion of the expression plasmid.** M: DNA marker DL10000. Lane 1: the product of eukaryotic expression plasmid pVAX1-14EGT digested by KpnI and XhoI restriction enzymes. Lane 2: the product of the prokaryotic expression plasmid pET-32a-14EGT digested by KpnI and XhoI restriction enzymes.

The selected fragments from the four common antigens, namely, 14–3-3, EF2, GAPDH, and transhydrogenase, were merged to create a chimeric gene named 14EGT. The chimeric gene 14EGT was inserted into both the eukaryotic expression vector pVAX1 and the prokaryotic expression vector pET-32a through ligation for expression in both eukaryotic and prokaryotic systems. For confirmation of the successful construction of the eukaryotic expression plasmid pVAX1-14EGT and the prokaryotic expression plasmid pET-32a-14EGT, restriction enzyme digestion and agarose gel electrophoresis were performed. The results of restriction enzyme digestion and agarose gel electrophoresis are shown in Figure 1. A band with a size of 2500 bp was observed in both Lane 1 (pVAX1-14EGT) and Lane 2 (pET-32a-14EGT), which corresponds to the expected size of the chimeric gene 14EGT. This finding indicates the successful construction of both expression plasmids. Additionally, the expression vectors pVAX1 (2900 bp) and pET-32a (5900 bp) were also found in Lane 1 and Lane 2, respectively. Subsequent gene sequencing analysis confirmed that the chimeric gene 14EGT was successfully inserted into both eukaryotic and prokaryotic expression plasmids, as there was 100% homology with the target gene.

### Purification and immunoblot analysis of the recombinant protein pET-32a-14EGT

**Figure 2:**
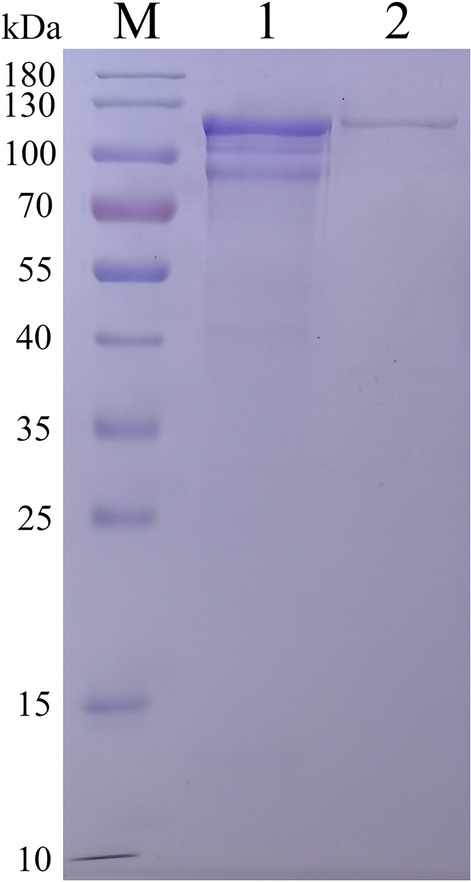
**Purification of the recombinant protein pET-32a-14EGT.** M: protein mid-molecular weight marker. Lane 1: the expressed proteins in inclusion bodies of the bacterial lysate before purification. Lane 2: the purified recombinant protein pET-32a-14EGT.

**Figure 3:**
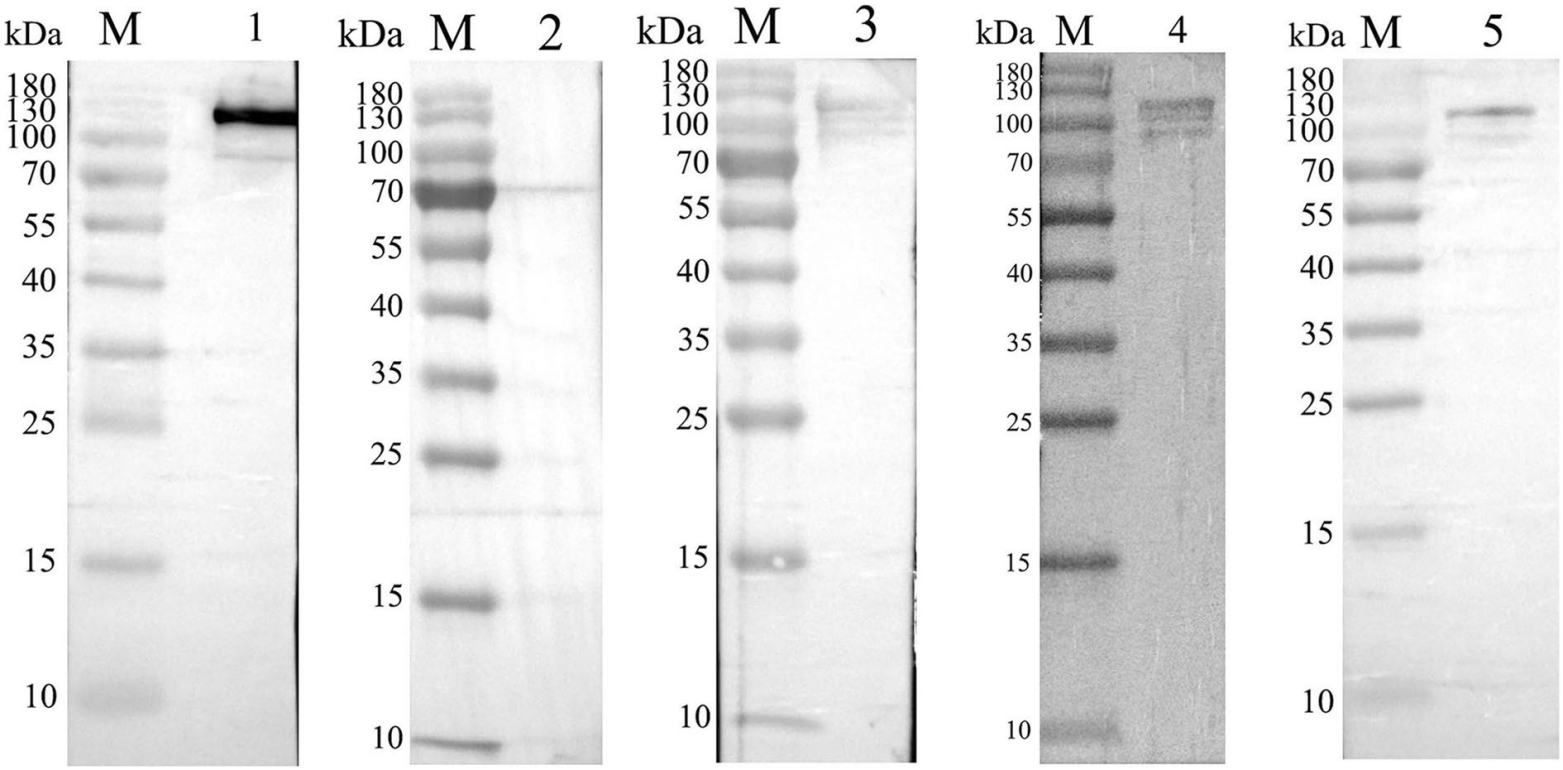
**Western blot analysis of the recombinant protein pET-32a-14EGT.** M: protein mid-molecular weight marker. Lane 1: The recognition of r14EGT by His-tag mouse monoclonal antibody. Lane 2: The recognition of r14EGT by normal chicken serum. Lane 3: The recognition of r14EGT by chicken serum against *E. tenella*. Lane 4: The recognition of r14EGT by chicken serum against *E. maxima.* Lane 5: The recognition of r14EGT by chicken serum against *E. acervulina*.

Following the strong expression of r14EGT using *E. coli* BL21, the expressed recombinant protein was purified using a Ni–NTA column. The results of protein purification are shown in Figure 2. Lane 1 shows the protein profile of the crude extract, while Lane 2 displays a single band of approximately 108 kDa, which corresponds to the expected molecular weight of r14EGT. This finding confirms the successful purification of the expressed recombinant protein. A Western blot analysis was performed to further confirm the identity of the purified r14EGT protein. His-tag monoclonal antibody and serum samples collected from chickens infected with *E. tenella*, *E. maxima*, and *E. acervulina* were used as primary antibodies to recognize the target protein (Figure 3). The results of the Western blot assays show that the purified r14EGT was recognized by both the His-tag monoclonal antibody and the anti-*Eimeria* species chicken serum, displaying a band of approximately 108 kDa. This finding indicates that the chimeric recombinant protein possesses good cross-reactivity among the three *Eimeria* parasites (*E. tenella*, *E. maxima*, and *E. acervulina*).

### Expression of pVAX1-14EGT in eukaryotic cells

**Figure 4:**
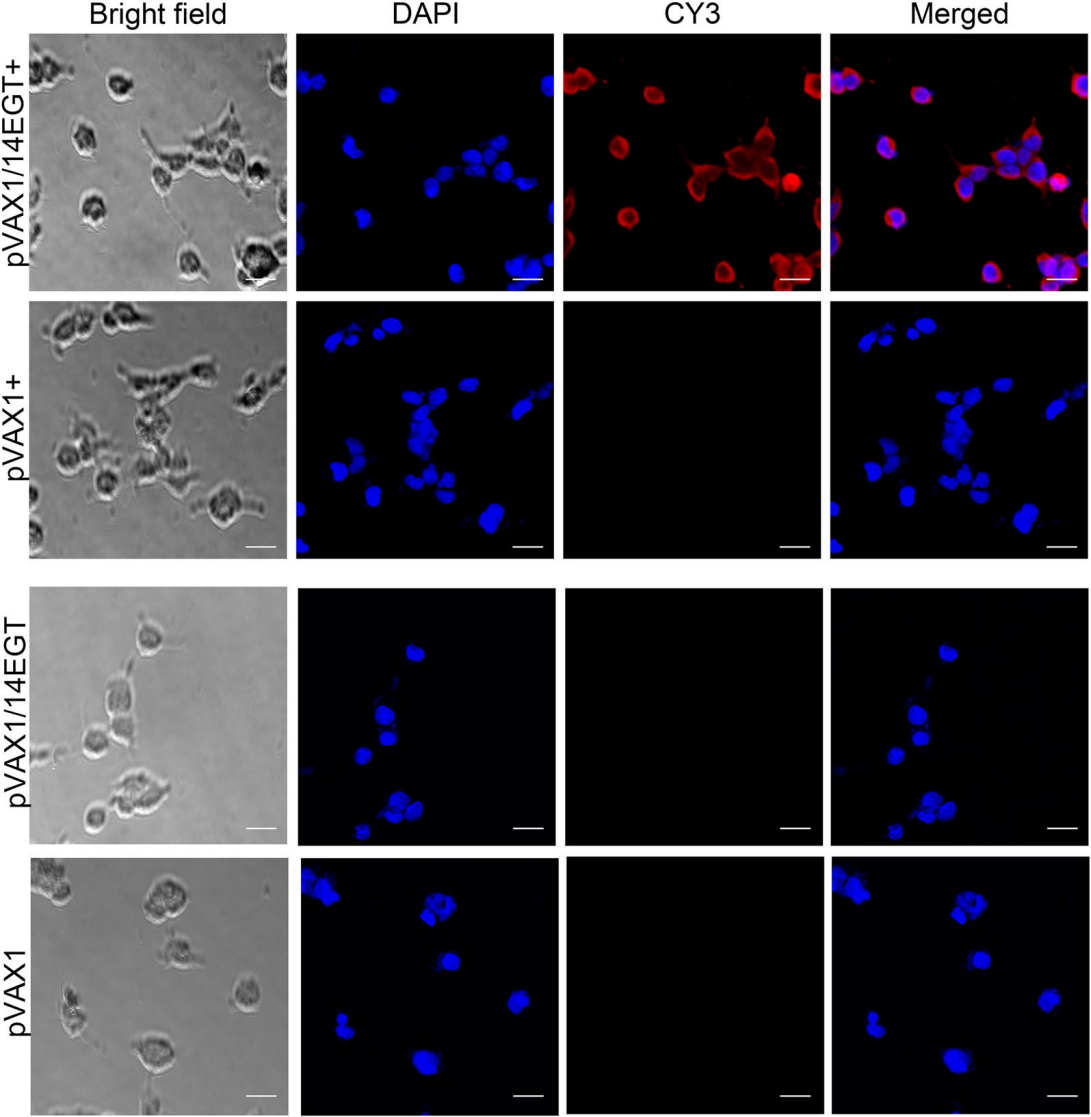
**Expression detection of pVAX1-14EGT in 293 T cells.** pVAX1/14EGT + : 293 T cells were transfected with the eukaryotic expression plasmid pVAX1-14EGT and detected by anti-r14EGT rat serum. pVAX1 + : 293 T cells were transfected with the eukaryotic expression vector pVAX1 and detected by anti-r14EGT rat serum. pVAX1/14EGT: 293 T cells were transfected with the eukaryotic expression plasmid pVAX1-14EGT and detected in negative rat serum. pVAX1: 293 T cells were transfected with the eukaryotic expression vector pVAX1 and detected in negative control rat serum. Bright field: view of the laser confocal microscope. DAPI: nuclear staining image. CY3: Cy3-labelled goat anti-rat IgG staining image. Merged: the merged graph of nuclear staining images and Cy3 staining images.

An immunofluorescence assay was conducted to determine the intracellular expression of the eukaryotic expression plasmid pVAX1-14EGT (Figure 4). In the bright-field images, the precise shape of the 293 T cells can be observed. Additionally, blue fluorescence, which corresponds to the position of the nucleus, appeared in all four groups stained with DAPI. Only the cells of the pVAX1/14EGT + group showed distinct red fluorescence, indicating that r14EGT was highly expressed in these cells and can be recognized by anti-r14EGT rat serum. These results confirmed the successful intracellular expression of the pVAX1-14EGT plasmid. In addition, the lack of red fluorescence observed in cells from the pVAX1 + , pVAX1/14EGT, and pVAX1 control groups further supports our hypothesis. In conclusion, our study confirms that the eukaryotic expression plasmid pVAX1-14EGT can be effectively expressed in eukaryotic cells.

### Immune responses induced by 14EGT vaccines in chickens

**Figure 5:**
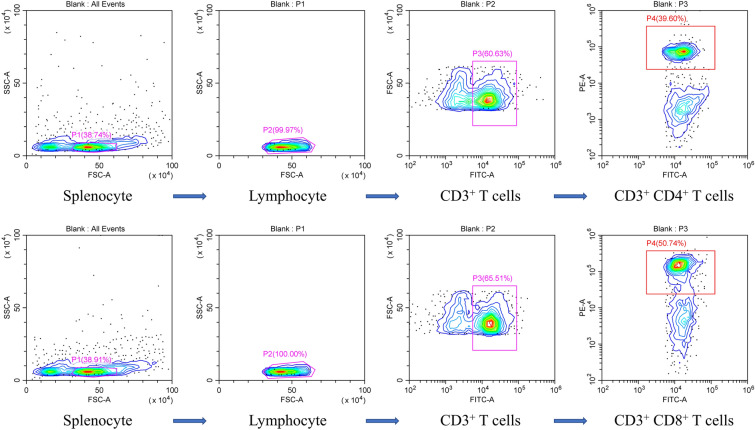
**The gating strategy for the analysis of splenocytes by flow cytometry.**

**Figure 6:**
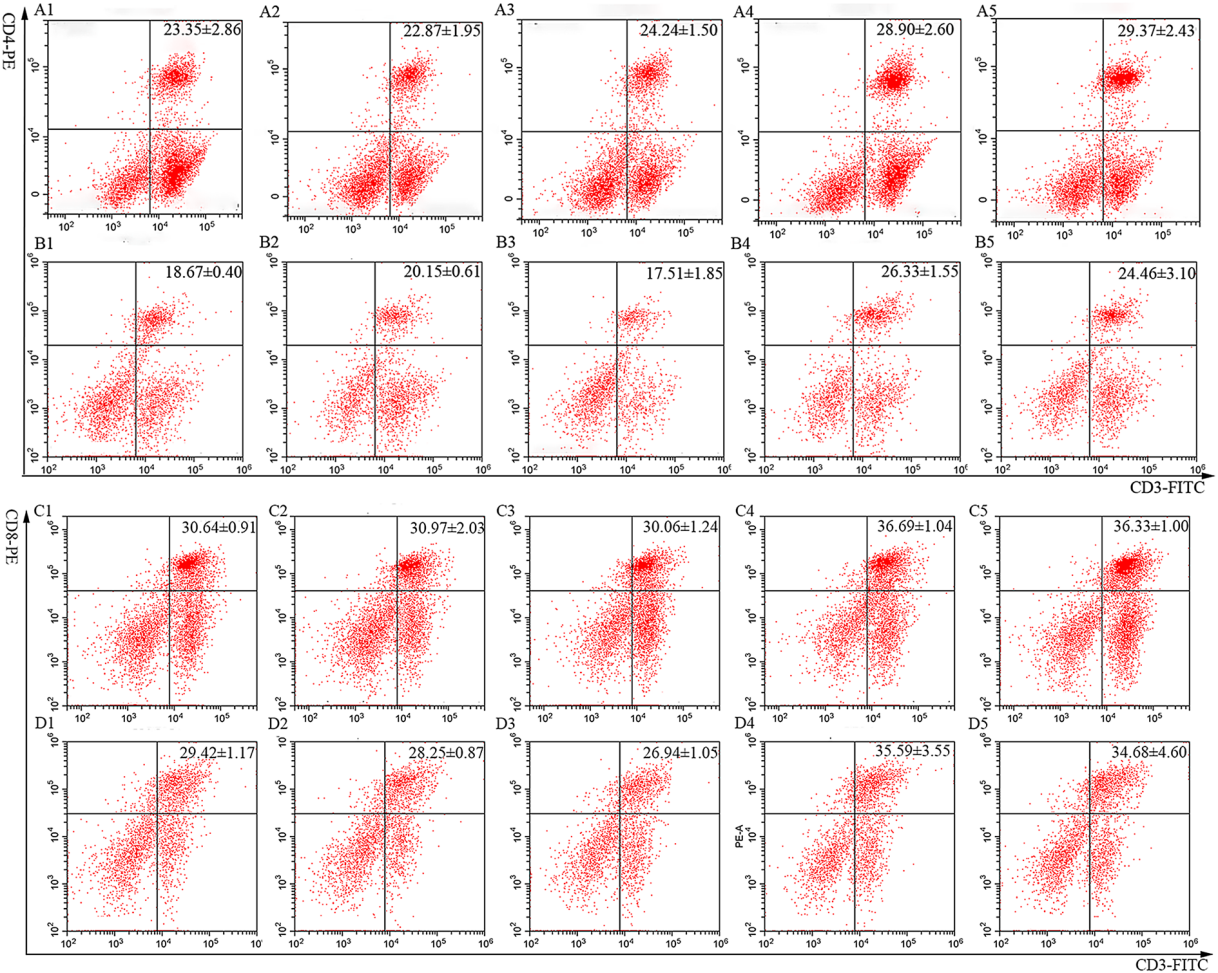
**The proportions of T lymphocyte subsets in the spleens of pVAX1-14EGT- and r14EGT-immunized chickens. A**: Determination of CD4^+^/CD3^+^ T lymphocytes in immunized chicken spleens at 21 days of age. **B**: Determination of CD4^+^/CD3^+^ T lymphocytes in immunized chicken spleens at 28 days of age. **C**: Determination of CD8^+^/CD3^+^ T lymphocytes in immunized chicken spleens at 21 days of age. **D**: Determination of CD8^+^/CD3^+^ T lymphocytes in immunized chicken spleens at 28 days of age. 1: PBS negative control. 2: The ratio of T lymphocytes in spleens from chickens immunized with pVAX1 plasmid. 3: The ratio of T lymphocytes in spleens from chickens immunized with pET-32a tag protein. 4: The ratio of T lymphocytes in spleens from chickens immunized with the eukaryotic expression plasmid pVAX1-14EGT. 5: The ratio of T lymphocytes in spleens from chickens immunized with recombinant protein pET-32a-14EGT.

**Figure 7:**
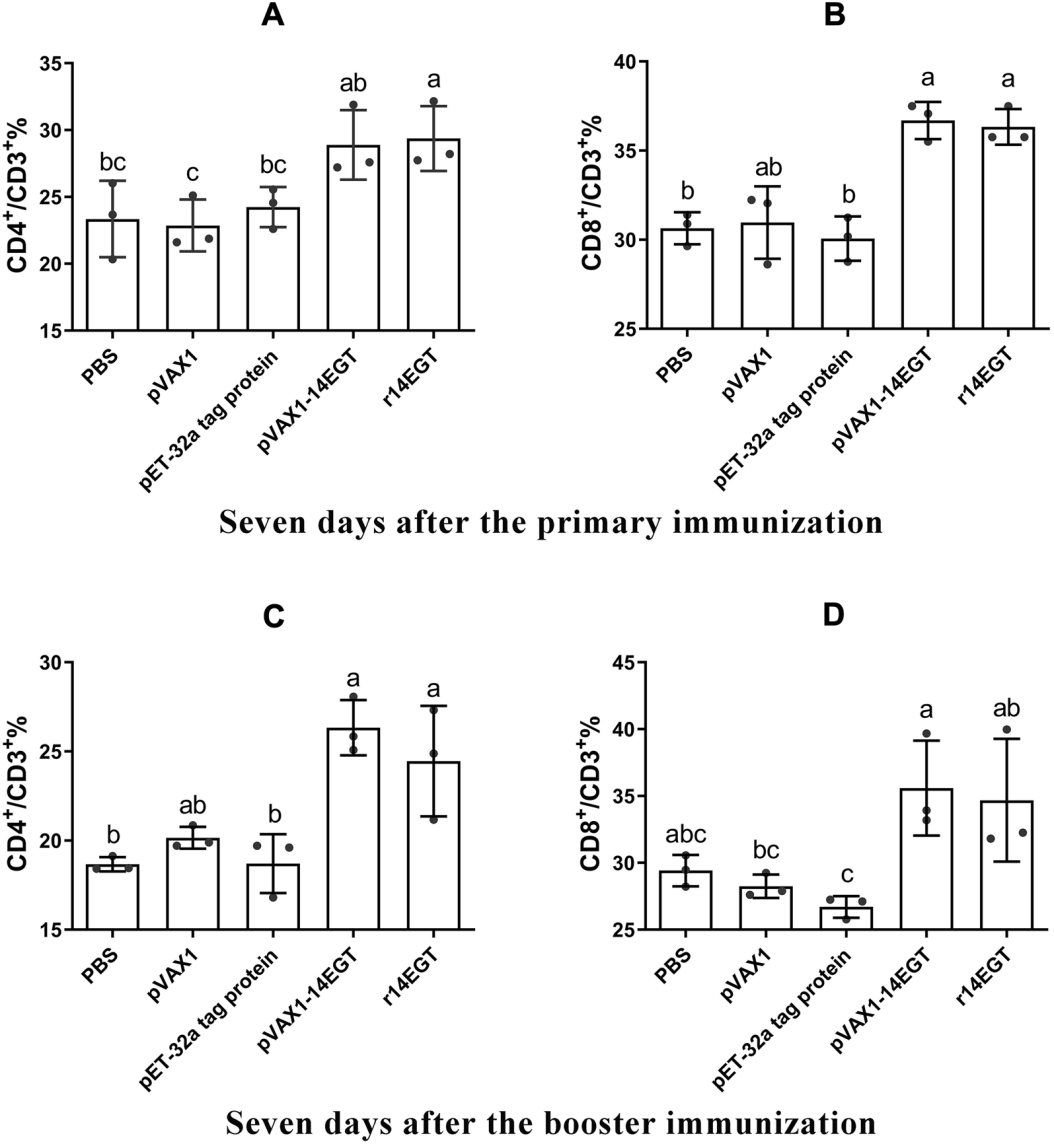
**The ratio of T lymphocyte subset in pVAX1-14EGT- and r14EGT-immunized chicken spleens. A** The ratio of CD4^+^/CD3^+^ T lymphocyte subset in immunized chicken spleen at 7 days after the primary immunization. **B** The ratio of the CD8^+^/CD3^+^ T lymphocyte subset in immunized chicken spleen at 7 days after booster immunization. **C** The ratio of CD4^+^/CD3^+^ T lymphocyte subset in immunized chicken spleen at 7 days after the primary immunization. **D** The ratio of the CD8^+^/CD3^+^ T lymphocyte subset in immunized chicken spleen at 7 days after booster immunization.

**Figure 8:**
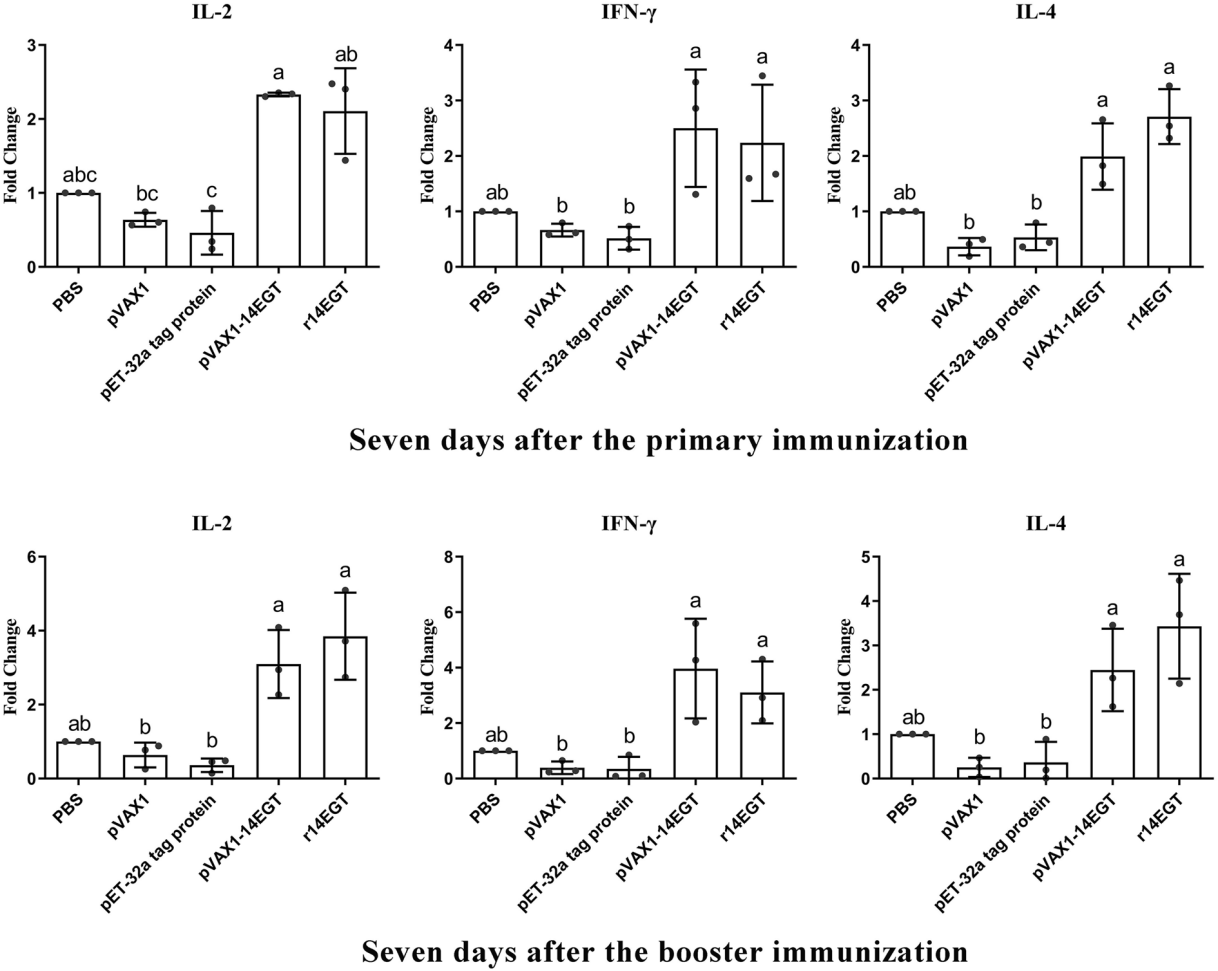
**Transcription levels of cytokines in splenocytes from chickens immunized with r14EGT and pVAX1-14EGT**.

Flow cytometry and qPCR were conducted to evaluate the cellular immune responses in chickens induced by vaccination with r14EGT and pVAX1-14EGT. Splenocytes were isolated from chickens seven days after the primary and booster immunizations. These splenocytes were utilized to measure the ratio change of T lymphocytes and the transcriptional levels of hallmark cytokines of Th1 and Th2 cells. The gating strategy employed for the analysis of splenocytes by flow cytometry is presented in Figure 5. From Figures 6 and 7, it can be observed that seven days after the primary immunization, the ratios of the CD4^+^/CD3^+^ T lymphocyte subset in the r14EGT group significantly increased compared to those in the control group (*p* < 0.05). A significant increase was observed in the ratios of CD4^+^/CD3^+^ T lymphocyte subsets in the pVAX1-14EGT experimental group compared to the pVAX1 control group (*p* < 0.05), while no significant difference was found when compared to the PBS control group (*p* > 0.05). Regarding the ratio of the CD8^+^/CD3^+^ T lymphocyte subset, the results in the r14EGT group demonstrated a significant increase compared to those of the control group (*p* < 0.05). While the ratio of the CD8^+^/CD3^+^ T lymphocyte subset in the pVAX1-14EGT group displayed a significant increase compared to that in the PBS group (*p* < 0.05), these differences were not significant when compared to the pVAX1 control group (*p* > 0.05). Seven days following booster immunization, the ratios of the CD4^+^/CD3^+^ T lymphocyte subset in the experimental groups were comparable to the ratios of the CD8^+^/CD3^+^ T lymphocyte subset observed seven days after primary immunization. The ratios of the CD8^+^/CD3^+^ T lymphocyte subset in both experimental groups exhibited significant differences from their empty vector or tagged protein control groups (*p* < 0.05). No significant difference was observed compared to the PBS control group (*p* > 0.05). Furthermore, no significant difference was observed among the PBS, pVAX1, and pET-32a tag protein control groups (*p* > 0.05). Seven days after the primary and booster immunizations, the transcription levels of IL-2, IL-4, and IFN-γ in the experimental groups exhibited significant differences when compared to those of the empty vector or tagged protein control groups (*p* < 0.05, as depicted in Figure 8). Compared with those of the PBS group, the levels of the three cytokines in both experimental groups showed an increase. However, the final data analysis indicated no significant difference between the experimental groups and the PBS control group (*p* > 0.05). Similarly, no significant difference was observed in the transcription levels among the control groups (*p* > 0.05). These results indicate that immunization with 14EGT vaccines effectively activates Th1/Th2 immune responses in chickens.

**Figure 9:**
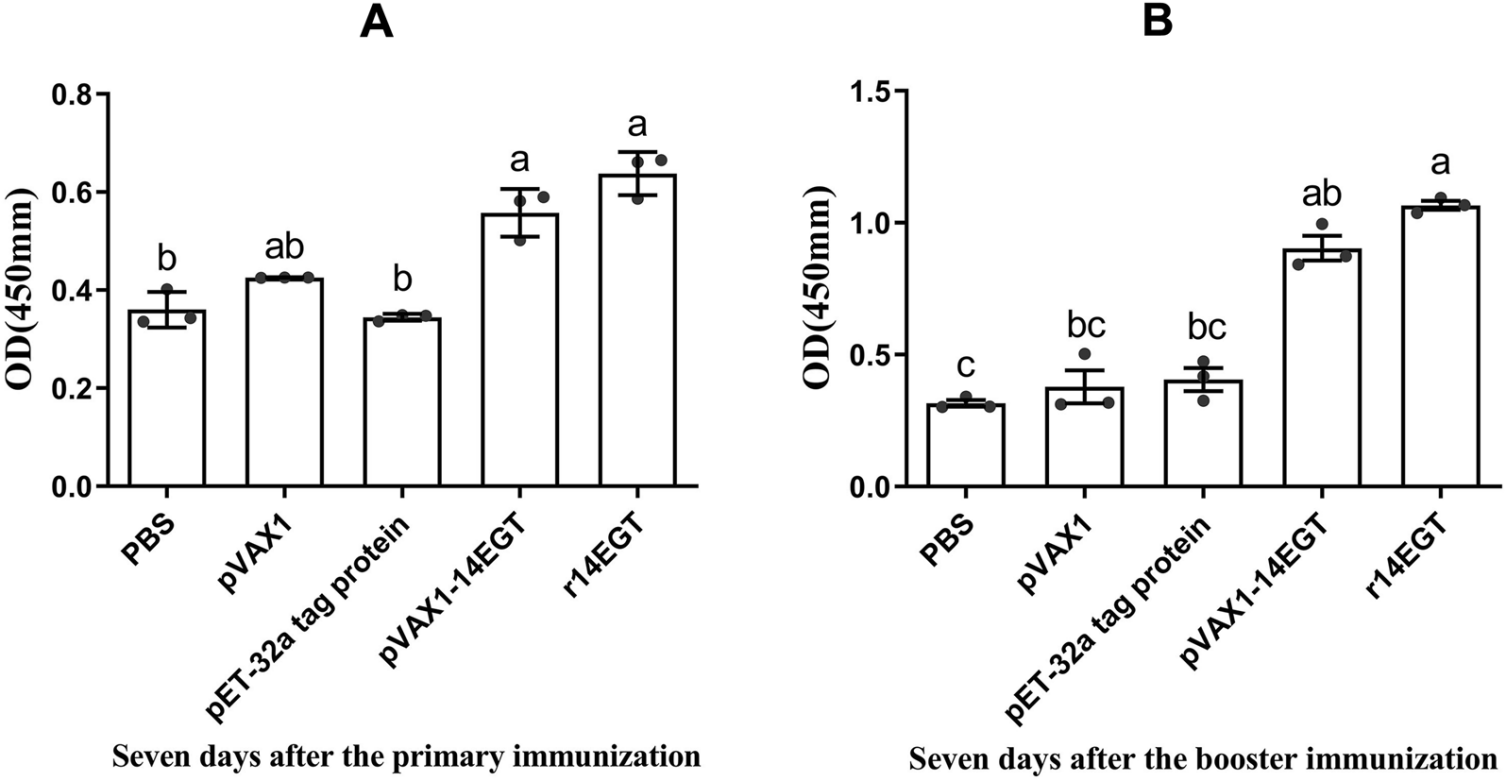
**Specific IgG titres in chicken serum induced by r14EGT and pVAX1-14EGT. A** Serum IgG titres from chickens at 7 days after the primary immunization. **B** Serum IgG titres from chickens at 7 days after booster immunization.

The levels of antigen-specific IgG antibodies in chickens immunized with 14EGT vaccines were determined by indirect ELISAs to assess the induced humoral responses (as shown in Figure 9). Seven days after the primary and booster immunization, the levels of specific antibodies in the r14EGT group exhibited a significant increase compared to those in the control groups (*p* < 0.05). Compared to the PBS group, the levels of specific antibodies in the pVAX1-14EGT group showed an increase. However, the final data analysis indicated no significant difference between the experimental group and the PBS control group (*p* > 0.05). In addition, no significant difference was observed in the transcription levels among the control groups (*p* > 0.05). The results showed that 14EGT vaccines could induce effective humoral immunity in the form of eukaryotic expression plasmids and recombinant proteins.

### Protective efficacy of 14EGT vaccines against challenge infection by *Eimeria* species

For evaluation of the protective efficacy of 14EGT vaccines, chickens were immunized with the vaccines and subsequently challenged with *E. tenella*, *E. maxima*, *E. acervulina*, or a mixture of oocysts from the aforementioned three *Eimeria* species. Body weight gains, oocyst shedding, enteric lesions, and ACI were used as indices to evaluate the protective efficacy of 14EGT vaccines. Based on the data presented in Table 4, a significant improvement in body weight gains was observed in the chickens immunized with 14EGT vaccines, regardless of whether the infection was caused by single or multiple *Eimeria* species. For the enteric lesions, the 14EGT vaccines significantly alleviated the damage caused by coccidiosis to the corresponding intestinal segments, except for *E. acervulina*. Furthermore, a significant decrease in oocyst output in faeces demonstrated the effective inhibition of pVAX1-14EGT and r14EGT on coccidian reproduction. The reduction rate of oocysts in faeces ranged from 62.17% to 82.04% when chickens were challenged with a single *Eimeria* species. However, a relatively low reduction rate of oocysts was observed in the chickens infected by multiple *Eimeria* species. The anticoccidial index reached a moderate level (160 < ACI < 180), indicating that the 14EGT vaccines have a moderate ability to protect chickens from the invasion of a single *Eimeria* species. However, unsatisfactory efficacies of the 14EGT vaccines were observed when chickens were challenged with mixed oocysts of *E. maxima*, *E. acervulina*, and *E. tenella*, resulting in ACI values of 142.18 and 146.41, respectively. Overall, the multiepitope DNA vaccine was found to be effective in protecting chickens from coccidiosis caused by a single *Eimeria* species. However, unsatisfactory protective efficacy of this vaccine against coinfections with multiple coccidian parasites was observed in this study.

## Discussion

With the continuous expansion of the global poultry industry, the impact of avian coccidiosis on chickens is also increasing. The estimated annual economic losses caused by *Eimeria* species are staggering, reaching USD 14.5 billion [40]. Careful husbandry and the use of anticoccidial chemicals may not effectively control coccidiosis for certain reasons. More practical and ideal strategies, such as immunoprophylaxis, are favoured by many researchers who are pursuing novel methods for controlling coccidiosis. Vaccination has been a long-standing effective control strategy for many diseases. Despite the fact that early live vaccines, to some extent, have reduced the impact of coccidiosis on the poultry industry, their potential for reverting to virulence and the difficulties associated with their production and transportation have made these traditional anticoccidial vaccines less promising for the future [41]. Recombinant protein vaccines and novel DNA vaccines encoding exogenous parasitic antigens have good potential for mass production, preservation, and transportation, which is a substantial improvement over traditional live vaccines. Moreover, these vaccines can provide safer and longer immune protection than conventional vaccines. Nevertheless, the primary challenge lies in identifying and selecting appropriate candidate antigens for vaccine construction. Screening epitopes of T or B cells from target antigens using bioinformatics methods to develop vaccines has emerged as a promising and time-efficient approach. Linking together selected epitopes in different orders to construct vaccines has been shown to induce robust immune protection against several parasitic infections. Examples of this approach are multiepitope DNA vaccines encoding surface antigens 1, 3, and 5, as well as rhoptry protein 8 from *T. gondii* [8, 9]. These vaccines have been shown to reduce parasite burden and extend the survival time of BALB/c mice. Majidiani et al. used a similar approach and screened epitopes binding to MHC and B cells from five *Toxoplasma* antigens [42]. These researchers then combined the selected epitopes to construct a multiepitope vaccine using *Leishmania tarentolae* as a delivery platform. Subsequent experiments aimed at assessing immune protection demonstrated that this multiepitope vaccine can elicit significant immune responses against acute toxoplasmosis. To control coccidiosis, Song et al. developed a multiepitope DNA vaccine containing a collection of concentrated T-cell epitopes that were screened from the merozoite antigen MZ5-7 and the sporozoite antigen SO7 [10]. Animal trials conducted thereafter confirmed the excellent protective efficacy of the vaccine in providing protection against *E. tenella* infection. In this study, DNAStar and NetMHC server software were used to develop a multiepitope anticoccidial vaccine. The vaccine was specifically designed to incorporate epitopes that bind to MHC molecules and T cells. We screened four common antigens using T-cell epitope density as the main index, complemented with antigenic index and hydrophilicity. Finally, we identified four fragments containing concentrated T-cell epitopes, a high antigenic index, and good hydrophilicity from these antigens. We subsequently analysed the binding sites and strength of these fragments to MHC molecules using NetMHCcons 1.1 Server and NetMHCIIpan 4.0 Server. As there is currently no mature tool available to predict antigen epitopes that bind to avian MHC molecules, the predictive analytics in this study were only performed by replacing chicken MHC molecules with human alleles that are highly similar to chicken MHC molecules [31, 32]. The T-cell binding epitopes were analysed by screening short peptides that bind to human MHC molecule alleles from the selected fragments. Similar studies were conducted by other groups [43, 44]. These researchers developed multiepitope vaccines targeting chicken anaemia virus and chicken *Mycoplasma gallisepticum* using the aforementioned screening methods. In this study, the results showed that all of the selected amino acid fragments (14, E, G, and T) contained epitopes that strongly bind to chicken MHC I and II molecules. Therefore, we selected these four amino acid sequences as the main components for constructing the multiepitope DNA and recombinant protein vaccines.

One of the challenges in developing an effective anticoccidial vaccine is that clinical coccidiosis infections are typically caused by more than one *Eimeria* species [11]. Therefore, antigens that exhibit high similarity across multiple *Eimeria* parasites hold promise as potential candidate antigens for vaccine development against coccidiosis [15]. Talebi previously discovered a conserved protein band with a molecular weight of 45 kDa in the sporulated oocysts of five major *Eimeria* species [16]. This conserved protein band exhibited successful reactivity with chicken serum against *E. maxima* in subsequent immunoserological experiments. Furthermore, Constantinoiu et al. and Sasai et al. identified an apical antigen that is highly conserved across various *Eimeria* species [17, 18]. Despite their immunodominant nature, these antigens have not been fully characterized through sequencing analysis, and their effectiveness in providing protection has yet to be evaluated. In an early study conducted in our laboratory, we identified five immunodominant antigens, namely, 14-3-3, EF-2, GAPDH, transhydrogenase, and ubiquitin-conjugating enzymes. These antigens showed more than 90% similarity across all seven chicken *Eimeria* species, with the exception of *Eimeria mitis* UCE [19]. Additionally, our subsequent studies have demonstrated that both 14-3-3 and GAPDH have displayed strong protective efficacy against *Eimeria* parasites, thus providing further confirmation of our initial screening results [20, 21]. Therefore, in this study, we selected four common antigens (excluding UCEs) to design and construct a multiepitope anticoccidial vaccine. As a significantly conserved protein family, 14-3-3 proteins possess crucial functions in various physiological and pathological mechanisms, including cellular immunity. These proteins trigger or interfere with the activity of specific protein partners to regulate various cellular processes [45]. Immunization with 14-3-3 protein has been shown to provide significant protection against *Schistosoma mansoni* and *T. gondii* in mice infected with apicomplexan parasites [46, 47]. Based on the report by Liu et al., the amino acid sequences of 14-3-3 protein in *E. tenella*, *E. maxima*, and *E. acervulina* share high similarities, with 97.5% similarity between *E. tenella* and *E. acervulina*, 99.6% between *E. acervulina* and *E. maxima*, and 99.1% between *E. tenella* and *E. maxima* [19]. In our previous study, the recombinant protein and DNA anticoccidial vaccines encoding the *E. acervulina* 14-3-3 protein demonstrated significant protective efficacy against single or multiple *Eimeria* species [20]. Therefore, the 14-3-3 protein holds promise as a key component in the fight against coccidiosis. Elongation factor 2 is highly conserved among the three main *Eimeria* parasites, with a pairwise similarity of 99%. In chicken coccidiosis, EF2 not only plays a role in the synthesis of proteins that function during the invasive stage but can also enhance the adaptability of parasites to the environment by regulating their own expression and activity. EF2 plays a dual role in chicken coccidiosis by participating in the synthesis of proteins essential for the invasive stage while also regulating the expression and activity of parasites, thus increasing their adaptability to the environment [48]. Another elongation factor, EF1α, which participates alongside EF2 in the protein synthesis elongation stage, has been shown to provide a certain level of cross-immunoprotection when facing infections by *E. tenella* or *E. maxima* [49]. EF1α is directly involved in the binding of aminoacyl tRNA to the ribosome, while EF2 facilitates the translocation of tRNA bound to mRNA through GTP hydrolysis [50]. Furthermore, the EF1α proteins from several common coccidia (*E. tenella*, *E. acervulina*, *E. maxima*, and *E. necatrix*) exhibit amino acid similarities exceeding 90%. Hence, although there is currently no research regarding the role of EF2 in resisting avian coccidiosis, we can infer from studies on EF1α that EF2 is likely to possess cross-immunity against various *Eimeria* species. Studies on *Leishmania*, *T. gondii*, and *Cryptosporidium parvum* have also revealed the characteristics of this molecule as a protective antigen against other apicomplexan parasites [51–53]. As a key enzyme in the process of glycolysis, GAPDH plays a vital role in several apicomplexan protozoa that rely almost entirely on glycolysis to sustain their life activities [54, 55]. Among the three *Eimeria* species, the amino acid sequence similarities of GAPDH are 94.1% between *E. tenella* and *E. acervulina*, 95.9% between *E. acervulina* and *E. maxima*, and 92.6% between *E. tenella* and *E. maxima*. In a previous study conducted in our laboratory, we found that a DNA vaccine encoding GAPDH from *E. maxima* and *E. acervulina* can induce significant cellular and humoral immune responses in chickens after immunization. These findings suggested the potential immune protection against coccidiosis provided by GAPDH [21]. Transhydrogenase exhibits amino acid sequence similarities of 93.4% between *E. tenella* and *E. acervulina*, 94.5% between *E. acervulina* and *E. maxima*, and 92.9% between *E. tenella* and *E. maxima* among the three *Eimeria* species. Studies have reported that transhydrogenase present in the *Eimeria* refractile body may be involved in ATP hydrolysis and respiration during sporulation [56]. Despite these findings, the physiological function of transhydrogenase in *Eimeria* parasites and its protective efficacy against coccidiosis require further investigation. In this study, the antigens mentioned above were utilized to design a multiepitope DNA vaccine, aiming to provide chickens with effective protection against mixed infections of *Eimeria* species.

Following invasion by *Eimeria* species in the host, cellular and humoral immune responses are activated. Over an extended period, the predominant defence against coccidiosis was believed to rely on the cellular immune response, primarily mediated by T cells [15]. The CD4^+^ and CD8^+^ T-cell subsets that participate in cellular immunity carry out distinct roles in combating infections caused by *Eimeria* parasites. Th1 cells, which differentiate from CD4^+^ T cells, secrete cytokines that play critical roles in combating infections. Recombinant interferon-γ, for instance, was found to stimulate target cells to produce multiple proteins that inhibit or limit the development of coccidia in host cells [24]. IL-2, a potent stimulatory factor for various immune cell populations, is commonly used as a cytokine adjuvant to enhance induced immune responses following vaccination [57, 58]. Th2 cells also differentiate from CD4^+^ T cells. Their hallmark cytokine, IL-4, is thought to be instrumental in inducing humoral immunity in the host [59]. Apart from CD4^+^ T cells, CD8^+^ T cells also assume critical roles in immune responses mediated by T cells. Infections caused by coccidiosis can trigger the differentiation of CD8^+^ T cells into cytotoxic T lymphocytes, which can specifically eliminate cells invaded by pathogens, thereby exerting protective effects on the host [25]. Furthermore, several reports have demonstrated a notable rise in the proportion of CD4^+^ and CD8^+^ T cells in chickens following infection by *Eimeria* species, providing evidence that these two T lymphocyte subsets participate in combating coccidiosis [60, 61]. Therefore, on the seventh day after the primary and booster immunizations with r14EGT and pVAX1-14EGT, the transcription levels of IFN-γ and IL-2, as well as IL-4, and changes in the ratio of CD4^+^ and CD8^+^ T lymphocytes were evaluated using qPCR and flow cytometry, respectively. The results indicate that there are significant differences in the transcription levels of three cytokines in the experimental groups compared to the corresponding empty vector or tag protein control groups. Additionally, the 14EGT vaccines also had a good promoting effect on the generation of CD4^+^ and CD8^+^ T-cell subsets. These findings suggest that immunization with 14EGT vaccines can effectively enhance strong cellular immunity and promote the proliferation and differentiation of T cells in vaccinated individuals. This effect is crucial for combating coccidiosis.

Although humoral immunity, which is mainly mediated by specific antibodies, was previously thought to play a minor role during coccidiosis, several recent studies have elucidated the correlation between humoral immune responses and coccidiosis [62, 63]. One convincing piece of evidence was provided by the first commercially available anticoccidial subunit vaccine, CoxAbic® (Netanya, Israel) [4]. It has been demonstrated that broiler offspring can acquire protection against coccidiosis from maternal antibodies produced by vaccinated laying hens. Similarly, Ding et al. reported a significant rise in the level of specific IgG antibodies in chickens immunized with vaccine constructs [64]. In the present study, we determined the level of antigen-specific IgG titres induced by immunization with r14EGT and pVAX1-14EGT. 7 days after the primary and booster immunization, the r14EGT vaccine significantly promoted the production of specific IgG antibodies in the serum. The effect of inducing humoral immunity by the pVAX1-14EGT vaccine is inferior to that of r14EGT. This finding could be because vaccines administered in the form of recombinant protein can more directly stimulate the humoral immune response. This result suggests that humoral immune responses may play a specific role in protecting against coccidiosis.

In this study, multiple criteria were employed to evaluate the protective efficacy of the constructed 14EGT vaccines, including enteric lesion, body weight gain, oocyst shedding, and ACI. As mentioned earlier, immunization with r14EGT and pVAX1-14EGT effectively improved the weight gain of chickens and reduced the number of oocysts shed in their faeces. However, there was no significant difference observed in the mean lesion score between the experimental groups and control groups when challenged with *E. acervulina*, in contrast to the findings in groups challenged with *E. tenella* or *E. maxima*. One possible explanation is that the enteric lesion induced by *E. acervulina* may not be as prominent as that caused by *E. tenella* or *E. maxima*. Therefore, any subtle differences in the invaded intestines between the experimental and control groups may not be easily discerned by the naked eye. In conclusion, after challenge with a single *Eimeria* species, the ACI of the experimental groups ranged from 162.4 to 173.17. These results suggest that the multiepitope DNA subunit vaccine 14EGT can provide chickens with moderate protection (ACI between 160 and 180) against coccidian infections caused by a single species. However, after exposure to mixed infections of *Eimeria* species, the r14EGT and pVAX1-14EGT vaccines showed limited efficacy. There may be several reasons for this outcome. *Eimeria* mixed infections typically result in more severe clinical symptoms than single *Eimeria* species infections due to their synergistic effects. This phenomenon may also be an important factor contributing to the suboptimal performance of the vaccine developed in this study when facing multiple *Eimeria* species infections. The optimal challenge dosage for mixed *Eimeria* species warrants additional evaluation based on insights from previous studies. Although intramuscular injection is considered to have better effects on triggering immunoprotection, improper procedures inevitably induce stress in animals, leading to potential deviations in results. Noninjectable immunization routes that are less likely to induce stress, such as mucosal water-drinking or spraying methods, are promising alternatives to intramuscular injection for large-scale clinical applications. An efficient and reliable vaccine delivery system is an indispensable part of the successful development of recombinant vaccines. Some probiotics, such as *Bacillus subtilis*, *Lactobacillus plantarum*, or *Lactococcus lactis*, have been shown to efficiently deliver vaccine antigens to recipients through noninjection immunization methods [65–68]. More importantly, these probiotics can enhance the intestinal microbiota ecosystem, immunity, and nutrient utilization and maintain the health of broiler chickens, thereby helping to reduce the adverse effects of pathogen infections. Appropriate adjuvants can also significantly enhance the immune protective effect of vaccines. Cytokine adjuvants such as IFN-γ and IL-2 have been reported to effectively improve the protective efficacy of the constructed vaccine [5, 6]. Emerging nano adjuvants also exhibit excellent enhancement to vaccines in a report of a multiepitope vaccine with PLGA nanospheres [69]. In addition, the optimal immune routes and doses for vaccine immunization need to be further explored to establish an optimal vaccination protocol. In conclusion, our study suggests that the use of bioinformatics methods to design multiepitope vaccines may be a practical strategy for combating the increasingly serious threat of coccidiosis.

### Supplementary Information

**Additional file 1: The prediction of concentrated epitopes of T cells from four common antigens using DNAStar Protean software.**

## Data Availability

All data generated or analysed in this research are included in this paper and its additional information files.
