# Supplementary material for: Protective efficacy of multiepitope vaccines constructed from common antigens of Eimeria species in chickens
Source: Vet Res. 2023 Dec 13;54:119. doi: 10.1186/s13567-023-01253-y (PMC10720236; doi:10.1186/s13567-023-01253-y)

**Additional file 1. The prediction of concentrated epitope of T-cell from four common antigens using DNAStar Protean software.**

**Additional file 1A:** The prediction of concentrated epitope of T-cell from 14-3-3


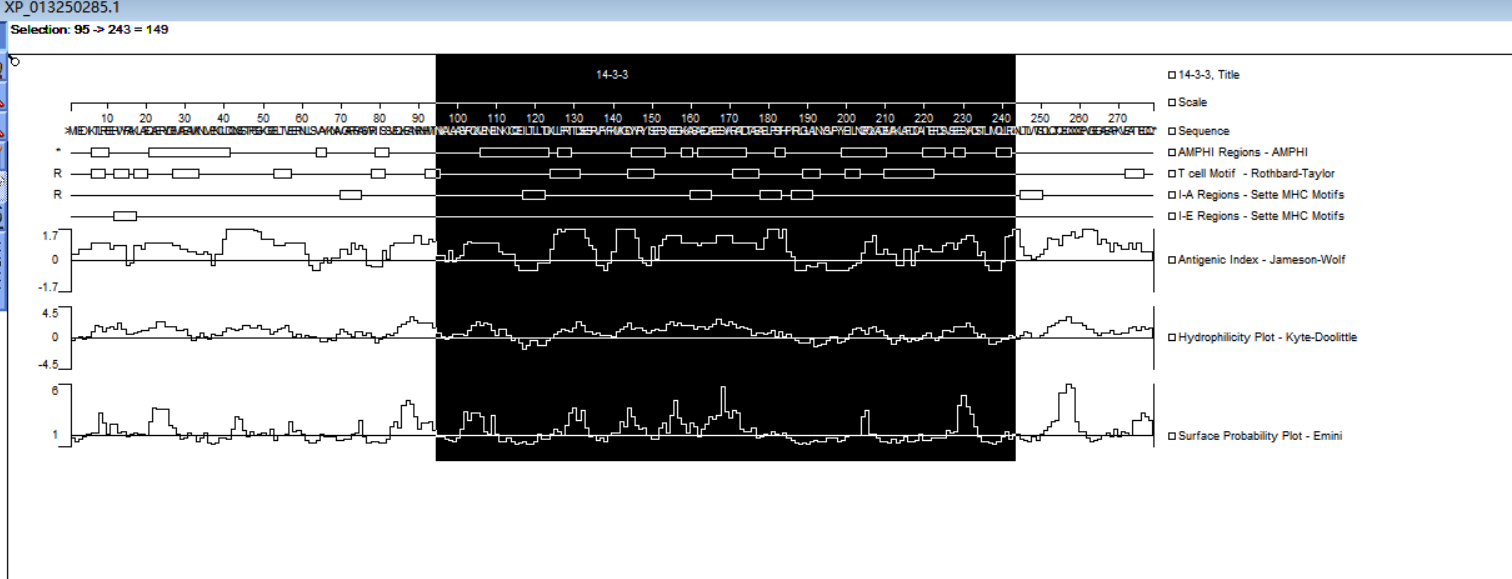


**Additional file 1B:** The prediction of concentrated epitope of T-cell from GAPDH


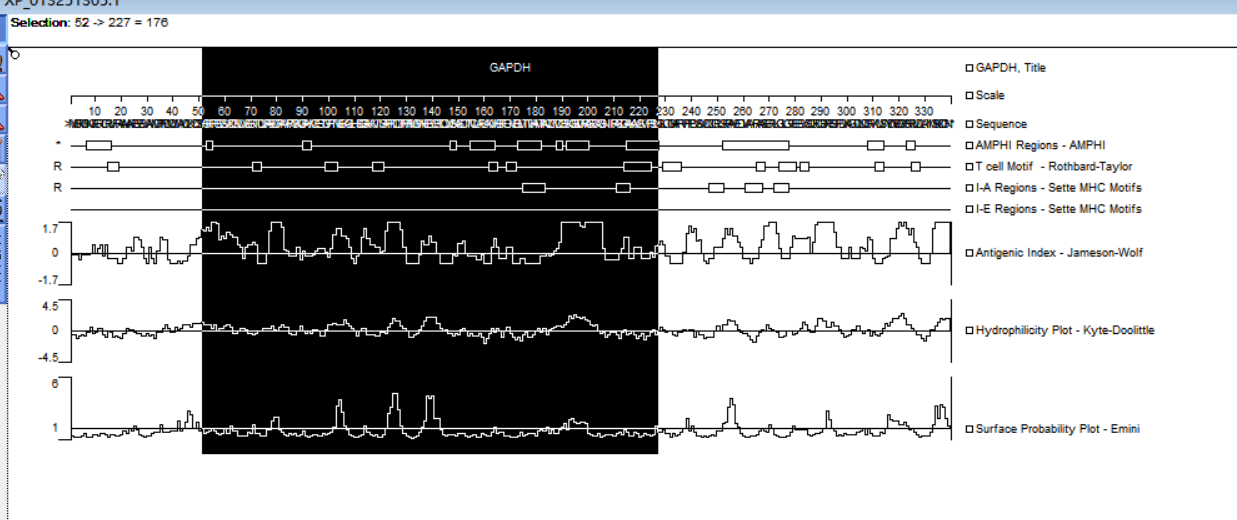


**Additional file 1C:** The prediction of concentrated epitope of T-cell from EF2


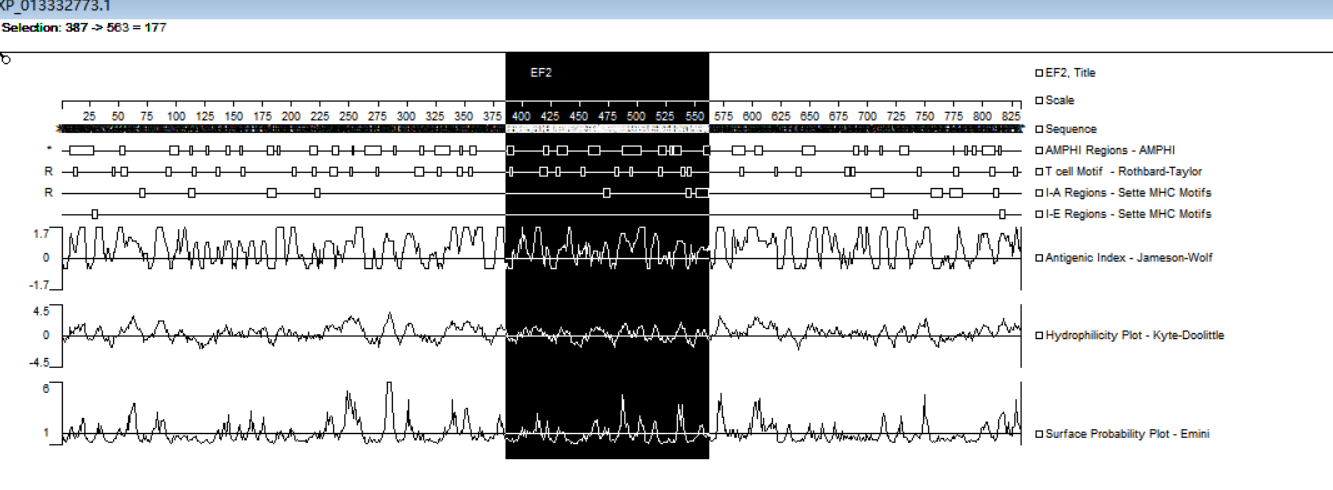


**Additional file 1D:** The prediction of concentrated epitope of T-cell from transhydrogenase


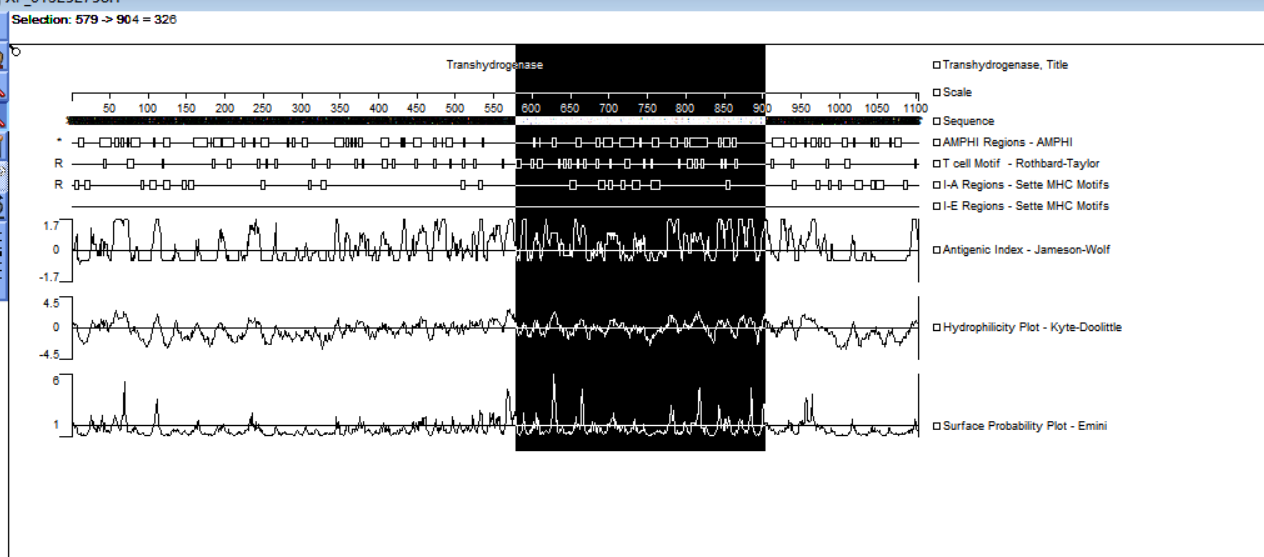

Supplement: Supplementary file 1 — Additional file 1: The prediction of concentrated epitopes of T cells from four common antigens using DNAStar Protean software. [file 13567_2023_1253_MOESM1_ESM.docx]
